# Enhanced delivery of carboplatin into brain tumours with intravenous Cereport^TM^ (RMP-7): dramatic differences and insight gained from dosing parameters

**DOI:** 10.1038/sj.bjc.6690450

**Published:** 1999-06

**Authors:** D F Emerich, P Snodgrass, R Dean, M Agostino, B Hasler, M Pink, H Xiong, B S Kim, R T Bartus

**Affiliations:** Alkermes Inc., 64 Sidney Street, Cambridge, MA, 01239, USA; Department of Pharmacology and Experimental Therapeutics, Tufts University Medical Center, Boston, MA, USA

**Keywords:** glioma, blood–brain barrier, chemotherapy, drug delivery, tumour barrier

## Abstract

Cereport^TM^ (RMP-7) is a selective bradykinin B2 receptor agonist which increases the permeability of the ‘blood–brain tumour barrier’ (BBTB) to increase delivery of chemotherapeutic agents to brain tumours. A series of experiments was performed in an RG2 rodent model of glioma to evaluate and refine intravenous (i.v.) parameters to optimize Cereport's clinical utility. The first experiment demonstrated that while carboplatin levels were increased by twofold when given as a bolus during the Cereport infusion, no increase in carboplatin levels were seen when Cereport and carboplatin were simultaneously co-infused for 15 min. A subsequent experiment established that a major factor responsible for the lack of an effect with the co-infusion paradigm was tachyphylaxis to Cereport during the 15 min infusion, for a progressively diminished response to Cereport occurred over that time frame, as plasma levels of carboplatin were rising. A final experiment adjusted the timing of the Cereport and carboplatin infusions so that higher plasma carboplatin levels were achieved prior to initiating the Cereport infusion. Significant uptake effects were achieved when the carboplatin infusion preceded the Cereport infusion by 10 min (i.e. 5 min overlap in the delivery of the two agents). Collectively, these data provide the first systematic evaluation of dosing parameters involving receptor-mediated changes in BBTB permeability and provide new information regarding the pharmacodynamics and potential clinical use of Cereport. © 1999 Cancer Research Campaign


*British Journal of Cancer* (1999) **80**(7), 964–970
© 1999 Cancer Research Campaign
Article no. bjoc.1998.0450

### Enhanced delivery of carboplatin into brain tumours with intravenous Cereport™ (RMP-7): dramatic differences and insight gained from dosing parameters

**DF Emerich[1], P Snodgrass[1], R Dean[1], M Agostino[1], B Hasler[1], M Pink[1], H Xiong[1], BS Kim[1] and RT Bartus[1,2]**

[1]Alkermes, Inc., 64 Sidney Street, Cambridge, MA 01239, USA; [2]Department of Pharmacology and Experimental Therapeutics, Tufts University Medical Center, Boston, MA, USA

**Summary** Cereport™ (RMP-7) is a selective bradykinin B2 receptor agonist which increases the permeability of the 'blood–brain tumour barrier' (BBTB) to increase delivery of chemotherapeutic agents to brain tumours. A series of experiments was performed in an RG2 rodent model of glioma to evaluate and refine intravenous (i.v.) parameters to optimize Cereport's clinical utility. The first experiment demonstrated that while carboplatin levels were increased by twofold when given as a bolus during the Cereport infusion, no increase in carboplatin levels were seen when Cereport and carboplatin were simultaneously co-infused for 15 min. A subsequent experiment established that a major factor responsible for the lack of an effect with the co-infusion paradigm was tachyphylaxis to Cereport during the 15 min infusion, for a progressively diminished response to Cereport occurred over that time frame, as plasma levels of carboplatin were rising. A final experiment adjusted the timing of the Cereport and carboplatin infusions so that higher plasma carboplatin levels were achieved prior to initiating the Cereport infusion. Significant uptake effects were achieved when the carboplatin infusion preceded the Cereport infusion by 10 min (i.e. 5 min overlap in the delivery of the two agents). Collectively, these data provide the first systematic evaluation of dosing parameters involving receptor-mediated changes in BBTB permeability and provide new information regarding the pharmacodynamics and potential clinical use of Cereport.

**Keywords:** glioma; blood–brain barrier; chemotherapy; drug delivery; tumour barrier

Gliomas represent a serious medical problem that continue to defy successful treatment. While surgery and radiotherapy generally add several months to a year of life to patients, the tumour inevitably recurs, most often leading to death within 6 months (Radhakrishnan et al, 1994). Chemotherapy has also proven generally unsuccessful in the treatment of gliomas. Despite major advances in the development of new chemotherapeutic agents, the treatment of gliomas has not been significantly impacted. At least one reason for the lack of significant effects of chemotherapy is the existence of the blood–brain tumour barrier (BBTB). Like the blood–brain barrier (BBB), the BBTB is comprised of vascular endothelial cells whose cytoarchitecture and closely packed arrangement preclude water soluble drugs from easily diffusing from the vessel lumen into the brain (tumour) space. While the BBTB is generally more leaky than the normal BBB, the barrier nonetheless persists in tumour vessels and represents a formidable obstacle to the delivery of potentially valuable drugs to brain tumours (Black, 1995).

Research extending over the past 2 decades has explored the concept that the permeability of the BBB and BBTB might be temporarily increased to deliver greater amounts of drugs into the brain and brain tumours respectively. This work was pioneered by investigators who infused hyperosmotic agents, such as mannitol and arabinose, directly into the carotid artery to temporarily disrupt the barriers (Neuwelt and Rapoport, 1984; Neuwelt et al, 1984*a*, 1985). These attempts were refined as investigators used endogenous peptides to increase the permeability of the vascular barriers by activating specific receptors expressed on the endothelial cells comprising the BBB and BBTB. The most noteworthy effort, to date, has occurred with the paracrine peptide, bradykinin (Wahl et al, 1987; Inamura & Black, 1994; Nomura et al, 1994). Most recently, Cereport™ (RMP-7) has been developed as the first receptor agonist to increase permeability of the BBB (Sanovich et al, 1995). Cereport is a bradykinin analogue which has a longer half-life and greater selectivity for the constitutively expressed B2 receptor (compared to bradykinin), thus providing two essential characteristics required of a drug intended to activate cerebral vascular receptors following parenteral administration (Doctrow et al, 1994; Straub et al, 1994; Bartus et al, 1996*b*).

Over the past several years, Cereport has been developed specifically as a means to increase delivery of chemotherapeutic agents to brain tumours. Testing in animal models of glioma (Inamura et al, 1994; Elliott et al, 1996*a*, 1996*b*, 1996*c*; Matsukado et al, 1996, 1998), as well as imaging studies in glioma patients (Warkne et al, 1995; Ford et al, 1996; Black et al, 1997) have demonstrated that Cereport not only increases permeability of the vasculature supplying brain tumours, but does so selectively. That is, the increased permeability is approximately 10–20 times greater in brain areas associated with tumours, as compared to normal, non-tumour brain (Bartus et al, 1996*b*). Recent phase II clinical trails in recurrent glioma offer preliminary evidence of patient benefit from Cereport when combined with carboplatin (Gregor et al, 1997; Prados et al, 1997; Chow et al, 1998) and a multinational phase III trial has been initiated for primary glioma.

*Received 23 June 1998*
*Revised 30 Novermber 1998*
*Accepted 7 December 1998*

*Correspondence to:* RT Bartus

The concept of using a receptor agonist such as Cereport to increase permeability of the BBTB, and thereby increase drug concentrations in brain tumours, represents a radical departure from the way in which brain tumours have been treated traditionally. Accordingly, a number of unique issues arise, because simply achieving the conventional 'maximum tolerated area under the curve (AUC)' of the chemotherapeutic drug may no longer yield optimal effects. Instead, attention must not only be given to the dose of both drugs administered but also, importantly, to the precise timing of their administration. These points become even more clear when one considers the complex pharmacodynamics involved with receptor-mediated modulation of the BBTB. For example, the increase in permeability induced by Cereport is transient, persisting for only about as long as the Cereport infusion lasts. In less than 2 min following the termination of Cereport infusions, restoration of the barrier occurs (Bartus et al, 1996*a*). Tachyphylaxis, or the gradual loss of pharmacological activity, has also been noted with intracarotid Cereport infusion, reflected by spontaneous restoration of the barrier within 30–60 min of continuous Cereport administration (Bartus et al, 1996*a*, 1996*b*). Thus, the precise timing of the Cereport infusion and that of the chemotherapeutic agent is crucial to whether any increased uptake into brain tumours will occur. However, the complex and transient nature of Cereport's effect on BBB permeability makes it difficult to predict what the optimal dosing paradigm might be. In principle, the time that the barrier is opened most widely should coincide with the peak plasma levels of the chemotherapeutic. Beyond that, knowing how quickly the barrier begins to open with Cereport, whether this response is graded or all-or-none, and precisely when tachyphylaxis first begins to occur will all influence the extent to which different combinations of dosing parameters enhance uptake of chemotherapeutic agents into the tumour.

To date, no systematic attempt has been made to address these issues with Cereport or any other receptor-mediated approach to modulating the BBTB. Thus, a series of experiments were conducted comparing different dosing combinations of Cereport and carboplatin in an attempt to further elucidate the pharmacodynamics involved with B2 receptor-mediated changes in BBTB permeability, and identify principles and dosing paradigms likely to be of benefit in the clinic.

### MATERIALS AND METHODS

### Rodent glioma model

#### *Subjects*

Male Fischer rats ($n$ = 286, 170–220 g; Taconic Farms, Germantown, NY, USA) were used in these studies. The rats were housed in pairs in polypropylene cages with free access to food and water. All procedures were reviewed and approved by Alkermes' Animal Care and Use Committee and were conducted in a manner which met or exceeded NIH standards.

#### *Tumour cell implantation*

Rat glioma (RG2) cells were maintained and implanted as previously described (Bartus, et al, 1996*a*, 1996*b*; Elliott et al, 1996*a*, 1996*b*). Briefly, rats were anaesthetized with a solution containing ketamine (24 mg ml$^{-1}$), xylazine (1.3 mg ml$^{-1}$) and acepromazine (0.33 mg ml$^{-1}$) and placed in a stereotaxic instrument. RG2 cells ($5 \times 10^4$ cells per 5 µl) were injected unilaterally into the striatum using a stereotaxic-mounted 10-µl Hamilton syringe with a 22-gauge needle at the following coordinates: A–P (+2.0 mm), L (+3.0 mm) and V (–6.5 mm) (Pellegrino et al, 1986).

### Dosing paradigm

#### *Blood vessel cannulation*

One week after tumour implantation, and under urethane anaesthesia (1.8 g kg$^{-1}$; intraperitoneally (i.p.), cannulae were placed in the jugular vein for drug administration and into both femoral arteries for the measurement of physiological parameters and the collection of blood used to calculate the uptake constant, $K_i$ (e.g. see Elliott et al, 1996*a*, 1996*b*).

#### *Drug administration and physiological monitoring*

Cereport (Alkermes, Inc., Cambridge, MA, USA) was dissolved in sterile 0.9% saline and infused intravenously (i.v.) using an infusion pump at a rate of 0.05 ml per min. Based on prior dose–response data collected with Cereport (Bartus et al, 1996*b*; Elliott et al, 1996*a*), the pharmacologically active doses of 4.5 and 9.0 µg kg$^{-}$ were used. [$^{14}$C]carboplatin (SA = 144 µCi mg$^{-1}$; Amersham, Arlington Heights, IL, USA) was given as either a bolus (100 µCi ml$^{-1}$ kg$^{-1}$) over 3 s or as a 15 min infusion into the jugular vein as described below (Figure 1). Throughout the experiment, body temperature was maintained normothermic (37.0 ± 1.0°C) and arterial blood gases, pH and blood pressure were monitored as previously described (Bartus et al, 1996*a*, 1996*b*; Elliott et al, 1996*a*, 1996*b*). Animals with physiological values outside the normal ranges (10–15% of all animals) were not used.

### Quantitative measurements

#### *Carboplatin pharmacokinetic plasma profile*

To determine the pharmacokinetic plasma profile of carboplatin, rats (approximately 225 g) were injected through the jugular vein cannula with either a bolus injection or 15 min infusion of [$^{14}$C]carboplatin (100 µCi kg$^{-1}$). Blood samples (300 µl) were taken via a femoral artery cannula during and following the radiolabel injection. Five animals received bolus injections of [$^{14}$C]carboplatin and had blood samples withdrawn at 1, 3, 5, 10, 15 and 20 min after the carboplatin injection. Sixteen animals received carboplatin infusions, and had blood withdrawn at 1, 3, 6, 9, 12, 24, or 30 min. To control for the possibility that the reduced blood volume would confound the AUC determinations, the total number of samples from individual animals ranged from 4 to 10 and was randomly distributed across the range of time sampling points. Accordingly, the total loss of blood ranged from 7.5% to 18% for individual animals. Radioactivity levels in the blood samples were determined using liquid scintillation counts. Following the determination of the plasma levels of radiolabelled carboplatin, the carboplatin AUC was determined for all animals using the linear trapezoidal method.

#### *Enhanced delivery of radiolabelled carboplatin*

For determination of carboplatin uptake into tumour tissue, arterial blood was withdrawn into PE90 tubing at a constant rate (0.04 ml min$^{-1}$) following administration of the radiolabel and prepared for scintillation counting, as previously described (Bartus et al, 1996*a*, 1996*b*; Elliott et al, 1996*a*, 1996*b*). At the end of the drug administration protocol, rats were decapitated, their brains rapidly removed, and the tumour was carefully dissected free. Tissue samples were weighed and incubated overnight at 40°C in

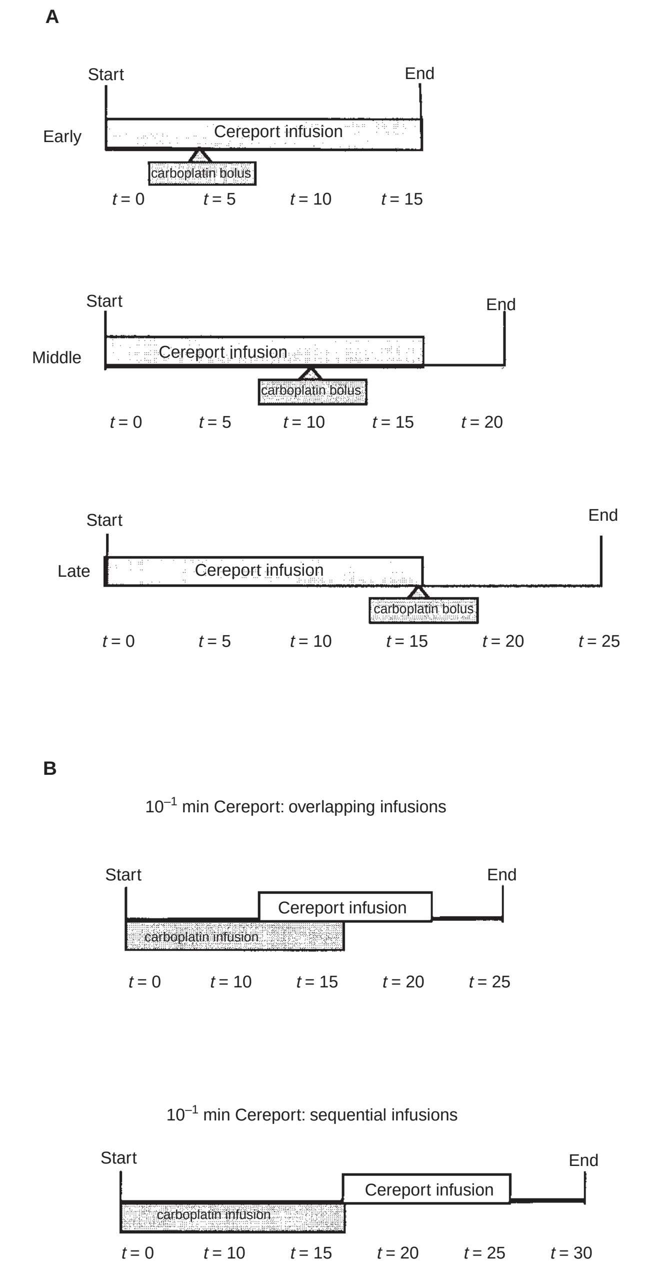

**Figure 1** **(A)** Schematic representation of the carboplatin infusion paradigm used to test the hypothesis that tachyphylaxis occurs during a 15 min i.v. infusion of Cereport. Animals received a 15 min i.v. infusion of Cereport and a bolus injection of [$^{14}$C]carboplatin at either 5 (early), 10 (middle), or 15 (late) min from the start of the Cereport infusion. All animals were sacrificed 10 min following the bolus injection of [$^{14}$C]carboplatin. (**B**) Infusion paradigms used to evaluate the effects of Cereport on [$^{14}$C]carboplatin levels when the Cereport and carboplatin infusions were overlapping (top) and sequential (bottom). All animals received a 15 min infusion of carboplatin. One group of animals (overlapping infusions) received a 10 min i.v. infusion of Cereport beginning 10 min into the carboplatin infusion. A second group of animals (sequential infusions) received Cereport beginning immediately at the end of the [$^{14}$C]carboplatin infusion. All animals were sacrificed 15 min following the initiation of the Cereport infusion

1 ml of Soluene. The following day, 10 ml of Hionic Fluor was added and radioactivity (nCi g$^{-1}$) was computed using scintillation counts. To account for differences in carboplatin concentration gradients under the various dosing paradigms used, the unidirectional transfer constant, $K_i$, was calculated as described earlier (Ohno et al, 1978; Ziylan et al, 1988), to provide an additional measure of the amount of [$^{14}$C]radiolabel taken up into tumour tissue.

### *Statistics*

Both the carboplatin pharmacokinetic plasma profiles and the effects of Cereport on delivery of carboplatin were compared in rats using a one-way analysis of variance (ANOVA) and post-hoc Dunnett's tests for multiple comparisons between control and treatment groups (JMP, SAS Institute Inc., Cary, NC, USA).

### RESULTS

### Bolus vs intravenous infusion of carboplatin

The initial experiment in this series compared the ability of i.v. infusions of Cereport to increase the levels of carboplatin in glioma, when carboplatin was given either as a bolus, or as a 15 min co-infusion (Figure 1). As shown in Figure 2, i.v. Cereport (9.0 μg kg$^{-1}$) increased carboplatin into the tumour by 75.5% ($P < 0.05$) when carboplatin was given as a bolus 5 min into the Cereport infusion. However, when Cereport and carboplatin were administered as 15 min co-infusions, no enhanced levels of carboplatin were seen at either of the two doses tested ($P > 0.1$).

Qualitatively, the carboplatin plasma profile achieved with the bolus was notably different from that achieved with a 15 min infusion. With the bolus injection, the peak plasma concentration or $C_{max}$ was higher, while the timing of its occurrence was earlier, as expected (Figure 3). However, when the AUC was calculated within the time frame that Cereport was infused, the two dosing paradigms produced AUCs that differed by less than 20% (bolus = 5745 ± 338 nCi; infusion = 4464 ± 61 nCi; $P < 0.01$).

### Test for tachyphylaxis

The ability of Cereport to increase tumour levels of carboplatin was dependent on the timing of the carboplatin bolus relative to the initiation of the Cereport infusion. A significant, 174% increase in carboplatin levels was seen when carboplatin was given 5 min into the Cereport infusion ($P < 0.01$). When the carboplatin bolus was given 10 min into the Cereport infusion, a significant ($P < 0.05$), but lesser effect of 112.4% was observed. Finally, when carboplatin was given 15 min from the initiation of the Cereport infusion, no increase in carboplatin levels was observed ($P > 0.1$), indicating that the barrier had restored (Figure 4). Thus, these data indicate that with constant i.v. infusions of Cereport, the effects on the BBTB exhibit tachyphylaxis (i.e. a gradual diminution in the pharmacological response over time).

### Refinement of dosing protocol

Contrary to the earlier co-infusion paradigm, Cereport enhanced tumour levels of carboplatin (by 142%; $P < 0.01$) when the infusion of Cereport was delayed and overlapped the carboplatin infusion by 5 min (Figure 5A). In contrast, when Cereport was administered only after the carboplatin infusion ended, the change in carboplatin tumour levels were negligible (i.e. less than 25%: Figure 5A). Similarly, when the data was converted to the unidirectional transfer constant ($K_i$) to account for possible effects of differences in plasma carboplatin concentrations, a significant increase in carboplatin delivery to tumour was observed in the

 © 1999 Cancer Research Campaign

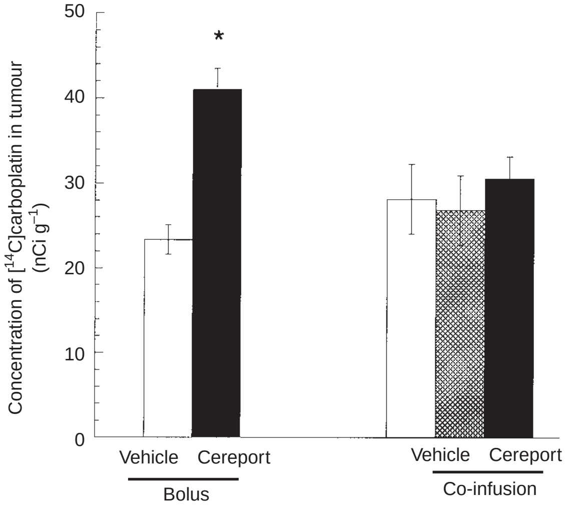

**Figure 2** Concentration of [$^{14}$C]carboplatin in tumour following a 15 min i.v. infusion of either saline or Cereport. The left panel represents the effects of Cereport (9.0 µg kg$^{-1}$) on carboplatin levels in tumour following a bolus injection of carboplatin given 5 min into the Cereport infusion (*n* for vehicle = 49, for Cereport = 50). The right panel depicts carboplatin levels following simultaneous co-infusions of vehicle (*n* = 20) or Cereport and carboplatin (Hatched bar = 4.5 µg kg$^{-1}$, *n* = 11; solid bar = 9.0 µg kg$^{-1}$, *n* = 14). Note that, in contrast to a bolus injection, co-infusions do not produce any effect on carboplatin levels in tumours. Data presented are mean ± s.e.m. nCi g$^{-1}$ tissue. *$P < 0.05$ vs saline

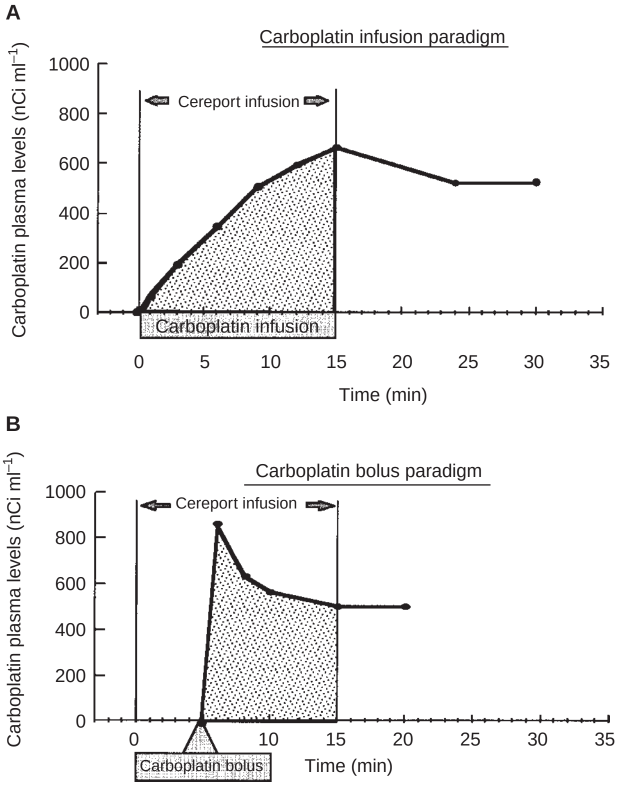

**Figure 3** Carboplatin plasma profile following infusions and bolus injections of [$^{14}$C]carboplatin. (**A**) The carboplatin plasma AUC (shaded area) following a 15 min i.v. infusion of labelled carboplatin together with a 15 min co-infusion of Cereport. (**B**) The AUC when the labelled carboplatin was administered as a bolus injection 5 min into the Cereport infusion

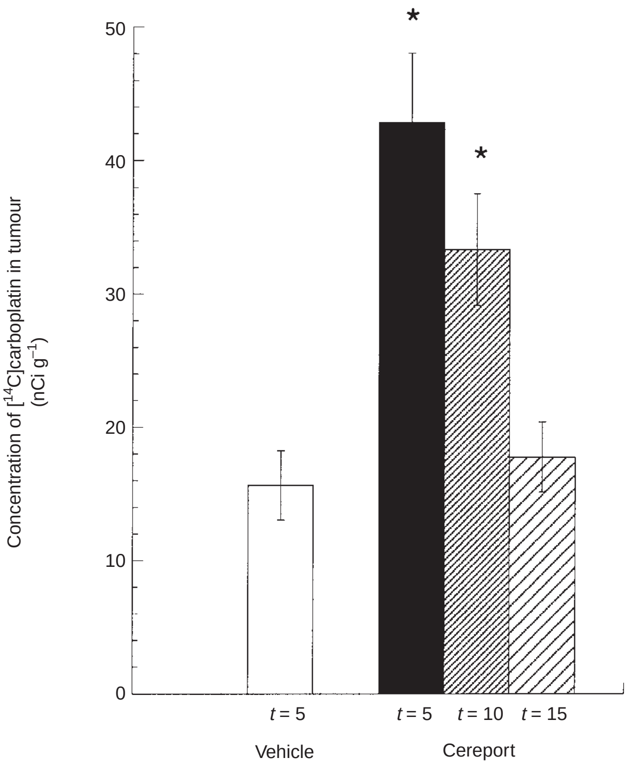

**Figure 4** Tachyphylaxis to a 15 min i.v. infusion of Cereport (9.0 µg kg$^{-1}$) using the paradigm outlined in Figure 1A. [$^{14}$C]carboplatin was administered as a bolus injection at 5 min into the saline infusion (*n* = 12) and 5 (*n* = 13) or 15 (*n* = 13) min into the Cereport infusion. Note that significant increases in carboplatin levels occur after 5 and 10, but not 15, min of continuous Cereport. Data presented are mean ± s.e.m. nCi g$^{-1}$ tissue. *$P < 0.05$ vs saline

**Table 1** Carboplatin plasma concentrations (nCi ml$^{-1}$ ± s.e.m.) under overlapping and sequential dosing conditions

| Condition | Overlapping paradigm | Sequential paradigm |
|---|---|---|
| Saline | 210.0 ± 8.21 | 214.0 ± 13.6 |
| Cereport | 269.0 ± 14.9* | 233.9 ± 9.90* |

Carboplatin plasma concentrations were calculated using blood obtained beginning at the start of the 10 min Cereport infusion and continuing for an additional 5 min. Cereport induced a modest increase in concentration in both the overlapping and sequential dosing conditions (*$P < 0.01$) vs saline-treated animals.

overlapping (65% increase; $P < 0.05$) but not the sequential dosing paradigm (Figure 5B).

An analysis of the plasma concentrations of carboplatin under the overlapping and sequential paradigms revealed a modest (< 20%) but significant increase in carboplatin concentrations in the Cereport group (Table 1; $P < 0.01$) relative to saline-treated animals. No significant interaction was observed between dosing condition (overlapping vs sequential) and Cereport ($P > 0.1$).

### DISCUSSION

Cereport represents the first compound in a new approach to treat malignant gliomas: receptor-mediated modulation of the BBTB to increase delivery of hydrophilic drugs to brain tumours. Previous

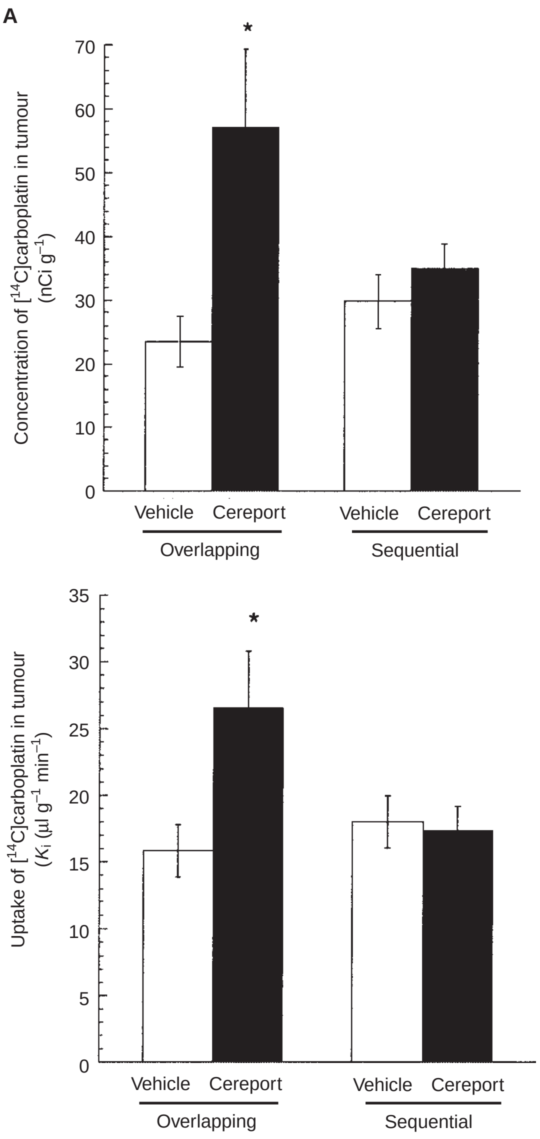

**Figure 5** **(A)** Effects of manipulating the timing and duration of Cereport and [$^{14}$C]carboplatin infusions as outlined in Figure 1B. All animals received 15 min i.v. infusions of labelled carboplatin together with a 10 min infusion of Cereport. A 10 min infusion of Cereport (9.0 µg kg$^{-1}$) increased carboplatin levels into tumours when its infusion overlapped the carboplatin infusion (left panels; $n$ = 18 for vehicle and 19 for Cereport) but not when the Cereport infusion followed the carboplatin infusion (right panels; $n$ = 16 for vehicle and 17 for Cereport). Data presented are mean ± s.e.m. nCi g$^{-1}$ tissue. *$P < 0.05$ vs. saline. (**B**) Representation of the same data as Figure 5A, but converted to unidirectional transfer constant ($K_i$) to account for possible effects of differences in plasma carboplatin concentrations. Note that the overlapping paradigm still produced a significant effect of Cereport, while the sequential paradigm did not. Data presented are mean ± s.e.m. $K_i$ (µl g$^{-1}$ min$^{-1}$) carboplatin work with animal models demonstrated that Cereport significantly increases uptake of carboplatin into brain tumours in a selective manner, in that the effect in non-tumour brain is 0.05 to 0.10 that of brain tumour-associated tissue (Bartus et al, 1996*b*). Evidence of Cereport's selectivity to tumour vasculature has been confirmed in the clinic using imaging techniques in glioma patients (Ford et al, 1996; Black et al, 1997). These preliminary clinical studies in human recurrent gliomas have also suggested that Cereport combined with carboplatin provides increased patient benefit when compared to carboplatin alone (Prados et al, 1997) or standard care (Gregor et al, 1997).

The experiments presented in this manuscript: (1) provide the first systematic evaluation of different i.v. dosing paradigms of Cereport; (2) contribute to the empirical foundation being built for receptor-mediated modulation of the BBTB; and (3) provide new information to aid in the design of future clinical trials and interpretation of the data collected. In summary, the results of these experiments demonstrate that i.v. infusions of Cereport significantly increase the concentration of carboplatin delivered to tumours, but these effects can vary dramatically, depending upon the specific temporal dosing parameters. Furthermore, these variations are due, in part, to the transient nature of the changes in BBTB permeability induced by Cereport.

### Tachphlyaxis as an important dosing issue

The initial experiment demonstrated that Cereport allowed significantly more carboplatin into the tumour when the latter was given as a bolus, but not when the two drugs were simultaneously infused over 15 min. This difference occurred despite relatively subtle differences in carboplatin plasma AUCs (i.e. < 20%) during the time that Cereport was infused (and therefore during the time permeability of the BBTB was increased) (Bartus et al, 1996*a*, 1996*b*). We hypothesized that the complete lack of an apparent effect in the co-infusion paradigm could be due to diminished permeability from i.v. Cereport during the latter portion of the 15 min, continuous infusion (i.e. that tachyphylaxis to Cereport's effects occur within the 15 min i.v. infusion). The second experiment in the series demonstrated that the permeability effects of Cereport do diminish during the 15 min infusion, in that the effects were greatest during the initial 5 min epoch, being approximately twofold higher than the values for the last 5 min epoch. These data support a classic, tachyphylaxis interpretation.

While this new evidence for tachyphylaxis is generally reminiscent of that reported previously with intracarotid Cereport (Bartus et al, 1996*a*, 1996*b*), the phenomenon reported here occurred several times more rapidly (e.g. in 15 min vs 60 min). The reason(s) for the difference in the rate of tachyphylaxis between studies are uncertain, but one salient difference is that the earlier studies used intracarotid infusions of Cereport, while the present study used i.v. infusions. With intracarotid Cereport, tachyphylaxis can be avoided or overcome if subsequent doses of Cereport are greater (Bartus et al, 1998). If the corollary is true, the lower Cereport concentrations at the cerebral vascular receptors that occur with i.v. dosing may permit tachyphylaxis to occur even more rapidly than is observed with intracarotid infusions. Consistent with this hypothesis is the observation that tachyphylaxis can be induced with low, near-threshold concentrations of Cereport (Bartus et al, 1996*a*). Alternatively, the increased rate of tachyphylaxis observed here may reflect subtle differences in this phenomenon when F344 rats, syngeneic to the RG2 tumour cells, are used (as was done in the present case), as opposed to when the outbred Wistar strain is used (as was true of the earlier, intracarotid studies). Further work will be required to confirm the difference observed and to test the hypotheses it suggests. Nonetheless, the tight, autoregulation of the permeability effect that is represented by tachyphylaxis further emphasizes the relative safety of a receptor-mediated approach to modulating the BBTB – a point also supported by direct animal (Riley et al, 1998) and human safety studies (Grous et al, 1996).

© 1999 Cancer Research Campaign

On the other hand, future work directed toward understanding and possibly delaying tachyphylaxis might reduce dosing restrictions that the phenomenon currently imposes. While the mechanism for Cereport tachyphylaxis is unknown, studies with bradykinin in other systems indicate that internalization and proteolytic degradation of the receptor occurs following its activation (Munoz and Lee-Lundberg, 1992). This response, and/or depletion of several second messengers associated with bradykinin signal transduction (including stimulation of G proteins, phosphoinositide turnover, prostaglandin response, and activation of both guanylate and adenylate cyclase) (Burch et al, 1993), are worthy of future investigations aimed at defining the mechanism of tachyphylaxis.

These data therefore emphasize a principle which may apply to all therapeutic approaches intended to increase delivery of chemotherapeutic drugs across the BBTB. That is, simply achieving the maximum tolerated AUC of the chemotherapeutic agent may produce disappointing results, for the precise timing of the $C_{max}$ and the surrounding slopes of the plasma curve must also be considered, relative to when permeability of the barrier is maximally increased. Accordingly, important prerequisites to employing this and similar receptor-based approaches to treating gliomas involve: (a) an accurate characterization of the plasma pharmacokinetic profile of the chemotherapeutic; (b) knowledge about the precise timing of the pharmacodynamics of the intended increase in permeability; and (c) a clinical dosing protocol that effectively incorporates this information by closely linking the timing of the two.

### Issues for refining Cereport dosing paradigms

These data demonstrate that increased delivery of carboplatin to tumour can be achieved following i.v. infusions of both Cereport and carboplatin, specifically when the infusion of Cereport is delayed until the plasma levels of carboplatin are substantially elevated. The data also offer further support for the hypothesis that simultaneous i.v. co-infusions of Cereport and carboplatin failed to produce significant increases in carboplatin levels because tachyphylaxis occurred during the 15 min Cereport infusion (i.e. prior to sufficient carboplatin plasma levels being achieved via co-infusion). However, what is not easy to explain is the lack of an effect when Cereport was administered at the end of the 15 min carboplatin infusion. The carboplatin pharmacokinetic plasma profile indicates that the AUCs during the Cereport infusion in the two dosing conditions are reasonably comparable (see Figure 3; $t$ = 10–25 min vs $t$ = 15–30 min). Thus, one would have expected the two dosing conditions to produce reasonably comparable carboplatin values in the tumour, but they did not.

Conceivably, Cereport might influence carboplatin protein binding in blood. However, tests of carboplatin protein binding in human blood (AV Boddy and HD Thomas, personal communication) and rat blood (unpublished observations) indicated that very little carboplatin binds to plasma proteins within the time frame of our experiments and that Cereport did not affect the extent of carboplatin protein binding.

Another possibility is that Cereport changed the plasma concentration of carboplatin (i.e. either increasing it during the overlapping portion of that paradigm, or decreasing it during the sequential paradigm). However, when the concentration of carboplatin was analysed in both the overlapping and sequential paradigms, only a modest (< 20%) increase in carboplatin concentration was observed in the Cereport groups with no significant difference between the two dosing conditions observed. Moreover, when $K_i$s were computed for the groups (thus factoring into the equation the different plasma concentration gradients), a significant difference in uptake effect of Cereport still existed between the two paradigms (Figure 5B). Thus, the small difference in carboplatin plasma concentrations cannot account for the difference in carboplatin delivery observed.

A third possible explanation involves the timing of the carboplatin $C_{max}$ (peak plasma concentration), relative to the Cereport infusion. In the overlapping dosing condition the $C_{max}$ occurred during the early part of the Cereport infusion, whereas in the sequential paradigm the timing of the $C_{max}$ had just passed as the Cereport infusion began. If an appreciable delay exists in the BBTB permeability effects of i.v. Cereport infusions, this might account for some of the difference observed between the two dosing paradigms.

While an explanation for these findings will require additional research, a number of salient points can be made about the small difference in carboplatin plasma levels observed: (a) they do not appear to exert a major influence on the uptake measures; (b) no evidence of increased toxicological liability has been observed in extensive human (Warkne et al, 1995; Grous et al, 1996; Gregor et al, 1997; Prados et al, 1997; Chow et al, 1998) and animal (Riley et al, 1998) studies; and (c) the phenomenon seems to be independent of renal clearance, given that the difference was observed within 10 min of Cereport, the fact that steady-state levels of carboplatin apparently existed, and that the plasma half-life of carboplatin is relatively long (i.e. 72 h).

Together, the data from this series of experiments with i.v. Cereport offer evidence that carboplatin delivery into gliomas can be enhanced when carboplatin is given as an infusion. The data also provide important insight into some of the essential dosing parameters required to use this method successfully, demonstrating the complex and transient nature of the vascular phenomenon exploited by this approach. They point to the importance of increasing the empirical data base to help in the design of new clinical dosing protocols, because entirely unexpected outcomes can result. Presumably, as results from additional research gradually accumulate, including the use of a wider range of chemotherapeutic agents, the empirical foundation supporting this novel approach to treating gliomas will be strengthened further, and the nuances underlying the approach will become increasingly clear. This should facilitate accurate predictions of the optimal dosing paradigms for different situations or circumstances. The systematic series of experiments reported here offers an essential step in that direction and emphasizes that the optimal condition for increasing uptake of carboplatin with Cereport includes initiation of the carboplatin infusion several minutes prior to Cereport, while maintaining a period of overlap in the two infusions.

### ACKNOWLEDGEMENTS

The authors acknowledge the technical assistance of Ms Patricia McDermott, Ms Joanne Marsh, Ms Denise Lafreniere and Ms Carroll Henschell in the conduction of the studies reported here, and the advice and assistance of Dr David Benziger in AUC determinations. The authors also appreciate and acknowledge the valuable comments of Mr Richard Pops, Dr Floyd Bloom, Dr Milton Brightman, Dr Stanley Rapoport and Dr Berislav Zlokovic on earlier versions of this manuscript and the assistance of Alexis Perkins in preparing the figures and manuscript for publication.

*© 1999 Cancer Research Campaign* 

### REFERENCES

Bartus RT, Elliott PJ, Dean RL, Hayward NJ, Nagle RL, Huff MR, Snodgrass PA and Blunt DG (1996*a*) Controlled modulation of BBB permeability using the bradykinin agonist, RMP-7. *Exp Neurol* **142**: 14–28

Bartus RT, Elliott PJ, Hayward NJ, Dean RL, McEwen E and Fisher SK (1996*b*) Permeability of the blood brain barrier by the bradykinin agonist, RMP-7: evidence for a sensitive, auto-regulated, receptor-mediated system. *Immunopharmacology* **33**: 270–278

Bartus RT, Snodgrass P, Dean R, Kim B, Black K and Emerich D (1999) Use of Cereport (RMP-7) to increase delivery of carboplatin to gliomas: insight and parameters for intracarotid infusion via a single-lumen cannula. *Drug Delivery* **6**: 15–21

Black KL (1995) Biochemical opening of the blood–brain barrier. *Adv Drug Rev* **15**: 37–52

Black KL, Cloughesy T, Huang S, Gobin Y, Zhou Y, Grous J, Nelson G, Farahani K, Hoh K and Phelps M (1997) Intracarotid infusion of RMP-7, a bradykinin analog, and transport of gallium-68 ethylenediamine tetraacetic acid into human gliomas. *J Neurosurg* **86**: 603–609

Burch R, Kyle D and Stormann T (1993) *Molecular Biology and Pharmacology of Bradykinin Receptors*. RG Landes: Austin

Chow KL, Cloughesy TF, Sayre JW, Gobin YP, Villablance JP and Vinuela F (1998) Factors predictive of response to selective intra-arterial chemotherapy. Conference Proceeding: American Soc Clin Oncol/Am Soc Neuro-Oncol: Los Angeles

Doctrow SR, Abelleira SM, Curry LA, Heller-Harrison R, Kozarich JW, Malfroy B, McCarroll LA, Morgan KG, Morrow AR, Musso GF, Smart JL, Straub JA and Turnbull B (1994) The bradykinin analog RMP-7 increases extracellular free calcium levels in rat brain microvascular endothelial cells. *J Pharmacol Exp Ther* **271**: 229–237

Elliott PJ, Hayward NJ, Dean RL, Blunt DG and Bartus RT (1996*a*) Intravenous RMP-7 selectively increases uptake of carboplatin into rat brain tumors. *Cancer Res* **56**: 3998–4005

Elliott PJ, Hayward NJ, Huff MR, Nagle TL, Black KL and Bartus RT (1996*b*) Unlocking the blood–brain barrier: a role for RMP-7 in brain tumor therapy. *Exp Neurol* **141**: 214–224

Elliott PJ, Hayward NJ, Dean RL and Bartus RT (1996*c*) Dissociation of blood–brain barrier permeability and the hypotensive effects of the bradykinin B2 agonist RMP-7. *Immunopharmacology* **33**: 205–208

Ford JM, Miles KA, Hayball MP, Bearcroft PW, Bleehen NM and Osborn CS (1996) A simplified technique for measurement of blood–brain barrier permeability using CT: preliminary results of the effect of RMP-7. In *Quantitative Imaging in Oncology*, Faulkner K (ed), pp. 126–155. Proceedings of the 19th LH Grey Conference. British Institute of Radiology

Gregor A, Lind M and Osborn C (1997) Intravenous RMP-7 and carboplatin: efficacy of a new treatment for recurrent malignant glioma. Conference Proceeding: Am Soc Clin Oncol/Am Soc Neuro-Oncol: Charlottesville, VA

Grous J, Elliott P and Bartus RT (1996) Development of RMP-7: a novel bradykinin agonist that increases delivery of chemotherapeutics to gliomas. In *Adjuvant Therapy of Cancer*

Inamura T and Black KL (1994) Bradykinin selectively opens blood–tumor barrier in experimental brain tumors. *J Cereb Blood Flow Metab* **14**: 862–870

Inamura T, Nomura T, Bartus RT and Black KL (1994) Intracarotid infusion of RMP-7, a bradykinin analog: a method for selective drug delivery to brain tumors. *J Neurosurg* **81**: 752–758

Matsukado K, Inamura T, Nakano S, Fukui M, Bartus RT and Black KL (1996) Enhanced tumor uptake of carboplatin and survival in glioma-bearing rats by intracarotid infusion of bradykinin analog, RMP-7. *Neurosurgery* **39**: 125–134

Matsukado K, Sugita M and Black KL (1998) Intracartoid low dose bradykinin infusion selectively increases tumor permeability through activation of bradykinin B2 receptors in malignant gliomas. *Brain Res* **792**: 10–15

Munoz CM and Lee-Lundberg LMF (1992) Receptor-mediated internalization of bradykinin. *JBC* **267**: 303–309

Neuwelt EA and Rapoport SI (1984) Modification of the blood–brain barrier in the chemotherapy of malignant brain tumors. *Fed Proc* **43**: 214–219

Neuwelt EA, Hill SA and Frenkel EP (1984) Osmotic blood–brain barrier modification and combination chemotherapy: concurrent tumor regression in areas of barrier opening. *Neurosurgery* **15**: 362–366

Neuwelt EA, Barnett PA, McCormick CI, Frenkel EP and Minna JD (1985) Osmotic blood–brain barrier modification: monoclonal antibody, albumin, and methotrexate delivery to cerebrospinal fluid and brain. *Neurosurgery* **17**: 419–423

Nomura T, Inamura T and Black KL (1994) Intracarotid infusion of bradykinin selectively increases blood-tumor permeability in 9L and C6 brain tumors. *Brain Res* **659**: 62–66

Ohno K, Pettigrew K and Rapoport S (1978) Lower limits of cerebrovascular permeability to nonelectrolytes in the conscious rat. *Am J Physiol* **253**: H299–H307

Pellegrino LJ, Pellegrino AS and Cushman AJ (1986) *A Stereotaxic Atlas of the Rat Brain*. Plenum Press: New York

Prados M, Schold H, Fine H, Jaeckle K, Hochberg F, Mechtler L, et al (1997) Randomized placebo controlled phase II study of i.v. carboplatin with or without i.v. RMP-7 in patients with recurrent malignant glioma. Conference Proceeding: Am Soc Clin Oncol/Am Soc Neuro-Oncol: Charlottesville, VA

Radhakrishnan K, Bohnen NI and Kurland LT (1994) Epidemilogy of brain tumours. In *Brain Tumors: A Comprehensive Text*, Morantz RA and Walsh JW (eds), pp. 23–32. Marcel Dekker: New York

Riley G, Kim N, Watson V, Gobin Y, LeBel C, Black K and Bartus RT (1998) Intra-arterial administration of carboplatin and the BBB permeabilizing agent, RMP-7: a toxicological evaluation in swine. *J Neuro-Oncol* **36**: 167–178

Sanovich E, Bartus RT, Friden PM, Dean RL, Le HQ and Brightman MW (1995) Pathway across blood–brain barrier opened by the bradykinin agonist, RMP-7. *Brain Res* **705**: 125–135

Straub JA, Akiyama A and Parmar P (1994) In vitro plasma metabolism of RMP-7. *Pharmaceut Res* **11**: 1673–1676

Wahl M, Unterberg A, Whalley ET, Bacthmann A, Young AR, Edvinsson L and Wagner FFW (1987) Effects of bradykinin on cerebral haemodynamics and blood–brain barrier function. In *Peptidergic Mechanisms in the Cerebral Circulation*, Edvinsson L (ed), pp. 166–190. Norwood: Chichester

Warkne P, Weyerbrock A and Ostertag C (1995) Effect of synthetic bradykinine RMP-7 on capillary permeability in glioblastoma multiforme. Cerebral Vascular Biology Conference: Paris.

Ziylan YZ, LeFauconnier JM, Bernard G and Bourre JM (1988) Effect of dexamethasone on transport of alpha-aminoisobutyric acid and sucrose across the blood–brain barrier. *J Neurochem* **51**: 1338–1342

 © 1999 Cancer Research Campaign
